# Endogenous and microbial biomarkers for periodontitis and type 2 diabetes mellitus

**DOI:** 10.3389/fendo.2023.1292596

**Published:** 2023-12-05

**Authors:** Songjun Li, Hongwen Li, Haiying Kong, Shang Ying Wu, Chak Kwong Cheng, Jian Xu

**Affiliations:** ^1^ Longgang Ear-Nose-Throat (ENT) Hospital, Institute of Ear-Nose-Throat (ENT) and Shenzhen Key Laboratory of Ear-Nose-Throat (ENT), Shenzhen, China; ^2^ Shenzhen Longgang Institute of Stomatology, Longgang Ear-Nose-Throat (ENT) Hospital, Shenzhen, China; ^3^ Department of Laboratory Medicine, Shenzhen Hospital, Peking University, Shenzhen, China; ^4^ School of Biomedical Sciences, Faculty of Medicine, The Chinese University of Hong Kong, Shatin, China

**Keywords:** biomarkers, periodontitis, diabetes mellitus, adipokines, microRNAs, oral microbiome

## Abstract

It has been well documented that there is a two-way relationship between diabetes mellitus and periodontitis. Diabetes mellitus represents an established risk factor for chronic periodontitis. Conversely, chronic periodontitis adversely modulates serum glucose levels in diabetic patients. Activated immune and inflammatory responses are noted during diabetes and periodontitis, under the modulation of similar biological mediators. These activated responses result in increased activity of certain immune-inflammatory mediators including adipokines and microRNAs in diabetic patients with periodontal disease. Notably, certain microbes in the oral cavity were identified to be involved in the occurrence of diabetes and periodontitis. In other words, these immune-inflammatory mediators and microbes may potentially serve as biomarkers for risk assessment and therapy selection in diabetes and periodontitis. In this review, we briefly provide an updated overview on different potential biomarkers, providing novel diagnostic and therapeutic insights on periodontal complications and diabetes mellitus.

## Introduction

**Figure 1:**
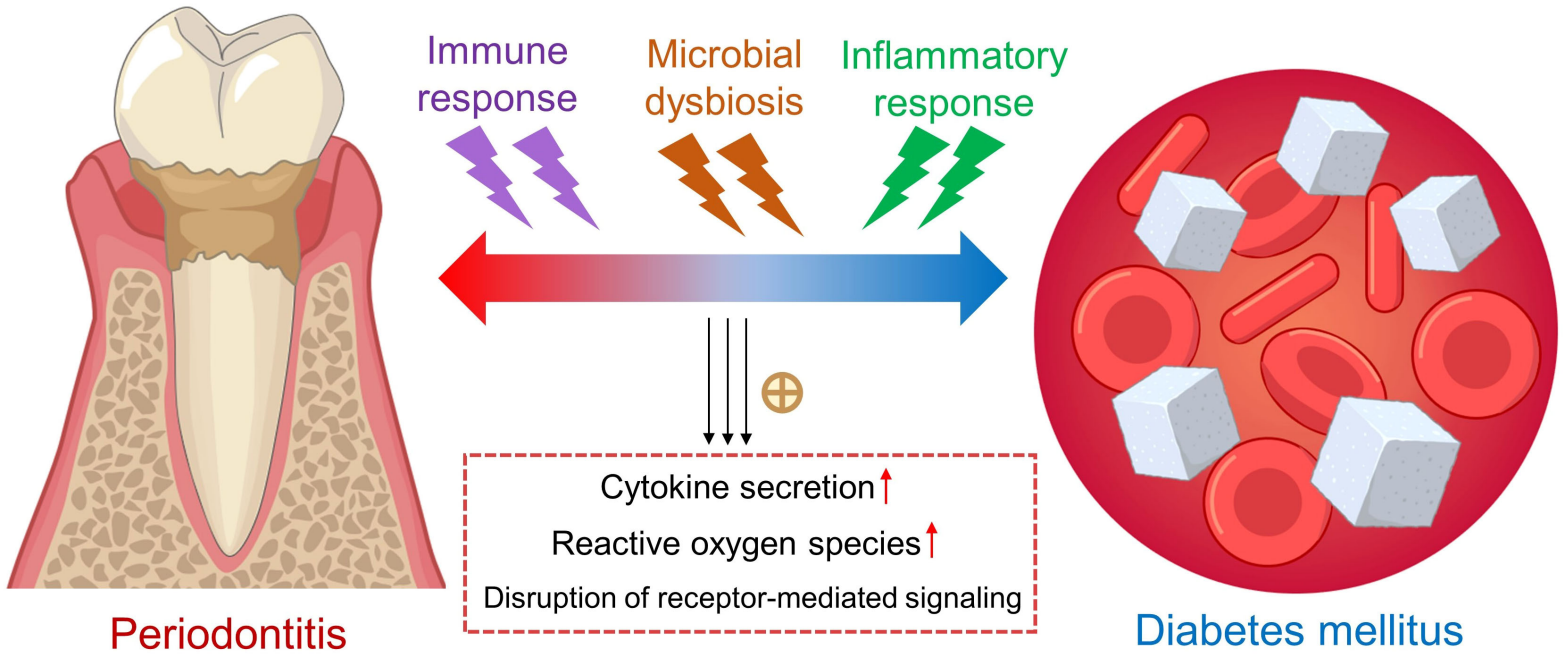
The bidirectional relationship between periodontitis and diabetes mellitus. Hyperactive immune and inflammatory responses participate in the vicious cycle between periodontitis and type 2 diabetes mellitus, associated with increased secretion of proinflammatory cytokines, higher oxidative stress and disruption of signaling pathways. Yellow cross: promotion. Up red arrows: increase.

Diabetes mellitus is clinically and genetically a heterogeneous group of disorders characterized by dysregulated nutrient metabolism, resulting from defects in insulin secretion and action (1). Hyperglycemia, the hallmark of diabetes mellitus, can lead to a range of chronic complications associated with long-term damage and dysfunction in various organs and body systems (2). Importantly, diabetic patients is more likely to develop chronic periodontitis (3), where a two-way relationship has been previously documented between diabetes and periodontitis (4, 5) (**Figure 1**). However, the detailed mechanisms underlying the bidirectional relationship remain largely unknown. Pathologically, the hyperactive inflammatory response plays a contributory role in the progression of these two diseases (6). Particularly, diabetes causes the activation of immune and inflammatory responses in periodontal tissues, increasing the risk of periodontitis. The activated responses subsequently result in increased secretion of cytokines, amplified oxidative damage, and disruption of receptor-mediated signaling. Altogether, these events accelerate the breakdown of periodontal connective tissues and resorption of alveolar bone, thus exacerbating periodontitis. In the other direction, periodontitis may cause dysregulated glycemic control in diabetic patients. Periodontal bacteria and their metabolic products, together with locally produced inflammatory cytokines and mediators in the inflamed periodontal tissues, enter the circulation to trigger systemic inflammation, further worsening glucose tolerance and insulin resistance (7). Certain pathogenic microbes in the oral cavity even aggravate the progression of periodontitis and diabetes mellitus by triggering host inflammation and causing burden on host immunity. Biomarkers serve as useful indicators during the onset and development of inflammatory and systemic diseases. In addition to the screening and risk assessment of diseases, biomarkers could also be applied in staging, grading, and selection of therapies (8). It has been reported that the levels of certain molecular biomarkers, such as adipokines and microRNAs, vary significantly in saliva, serum and gingival crevicular fluid (GCF) of individuals with both diabetes mellitus and periodontitis (9). Moreover, several strategies have been developed to safely and conveniently collect GCF from individuals, such as extracrevicular and intracrevicular GCF collection techniques (10). Hence, saliva, serum and GCF represent feasible sources of biomarkers for diagnostic purposes. This article aims to review the potential biomarkers in diabetes and periodontitis, providing novel diagnostic and therapeutic insights on periodontal complications in associated with diabetes mellitus.

## Adipokines

Adipokines are a group of secretory proteins mainly released by an active endocrine organ, particularly adipose tissue, into the systemic circulation. Adipokines are believed to be tightly associated with energy control, insulin sensitivity, and immune-inflammatory responses (11, 12). During diabetes mellitus, abnormal metabolism in adipose tissue may affect various organs via adipokine production (13). Periodontitis can lead to a proinflammatory state and affect adipokine levels in serum, tear fluid and GCF of obese patients (14, 15). Furthermore, diabetes mellitus can promote dyslipidemia and inflammation via regulation of adipokines (16). It has been postulated that the concentration of adipokines might be indicative to chronic metabolic disorders and pathological processes in local tissues. Therefore, any variations of adipokine levels in body fluids might be indicative to the severity of diabetes mellitus and chronic periodontitis (11) (**Table 1**).

**Table 1:** Adipokines as potential biomarkers for periodontitis and diabetes mellitus.

Adipokines	Biological functions	Correlation with periodontitis	Correlation with diabetes mellitus	Ref
Adiponectin	· Exerts anti-inflammatory effects · Regulates glycemia · Acts as endogenous insulin sensitizer	· Periodontal treatment increases its serum level	· Inversely correlated with obesity and insulin resistance	(17–20)
Resistin	· Induces insulin resistance in animals · Aggravates inflammation in humans · Stimulates the secretion of proinflammatory cytokines (e.g. TNF-α, IL-6 and IL-12)	· Positively correlated with periodontitis	· Positively correlated to proinflammatory cytokine levels during diabetes mellitus · Increased GCF level of resistin in diabetic patients with chronic periodontitis	(21–26)
Visfatin	· Reduces glucose release · Stimulates glucose utilization in adipocytes and monocytes · Stimulates the production of proinflammatory cytokines (e.g. TNF-α, IL-6 and IL-1β)	· Positively correlated with periodontitis · Non-surgical periodontal treatment reduces its serum and GCF levels	· Positively correlated to proinflammatory cytokine levels during diabetes mellitus · Positively correlated with diabetes-related periodontitis	(27–31)

*Red color: body fluids or tissues/cells where these biomarkers could be collected for potential diagnostic purpose.

### Adiponectin

A bidirectional relationship exists between diabetes mellitus and periodontitis (32). It has been reported that diabetic subjects with insulin resistance are more likely to develop severe periodontitis (33, 34). Adiponectin, an adipokine mainly secreted by adipocytes, exerts anti-inflammatory effects and plays a pivotal role in regulating glycemia (17). Adiponectin acts as an endogenous insulin sensitizer in the regulation of insulin sensitivity, and its level is inversely correlated with obesity and insulin resistance (18). Some intervention studies have suggested that periodontal treatment could significantly increase serum adiponectin levels in type 2 diabetic patients with periodontitis (19, 20). Furthermore, periodontal therapy has been shown to be associated with improved glycemic control and insulin resistance in diabetic patients (35). More importantly, another clinical trial stated that periodontal intervention also improved lipid profile, reduced inflammatory cytokines in serum, and elevated levels of serum adiponectin in diabetic patients (34). Adiponectin profoundly improves insulin sensitivity by inhibiting glucose output from the liver, and limiting glucose uptake by adipose tissue and muscle (36).

### Resistin

Named after its apparent ability to ‘*resist insulin*’, resistin is another adipokine first discovered in murine. Resistin is an 11 kDa protein encoded on chromosome 8, which was once classified as a unique signaling molecule in-between obesity and type 2 diabetes mellitus (21, 26). In multiple obese animal models, resistin was shown to induce insulin resistance, where hyper-resistinemia remarkably impairs insulin sensitivity. However, the exact role of resistin in obesity and type 2 diabetes mellitus in humans have not been comprehensively defined (37–39). In contrast, human resistin is predominantly expressed in peripheral-blood mononuclear cells. It aggravates inflammation which has been conclusively associated with the development of obesity and insulin resistance (23). It has been reported that resistin acts as a proinflammatory molecule by stimulating the secretion of tumor necrosis factor α (TNF-α), interleukin (IL)-6 and IL-12, thereby inducing its own production via a positive feedback cycle during periodontitis (22). Of note, the elevated levels of resistin in periodontal disease may highlight its role as a specific and sensitive biomarker in the early detection and intervention of diabetes-related periodontitis. Joshi et al. has reported that GCF level of resistin was significantly higher in diabetic patients with chronic periodontitis, where its level showed no correlation with glycated hemoglobin (HbA1c) value (24). Such finding indicated that resistin is more closely related to the inflammatory condition instead of the glycemic state of the individual, suggesting resistin as an inflammatory biomarker in two diseases. Another study has shown that single-nucleotide polymorphism of resistin gene is correlated to the resistin levels in serum and GCF of diabetic patients with chronic periodontitis (25). Moreover, *in vitro* clues are present that release of inflammatory cytokines, such as IL-6 and TNF-α, and maturation of monocytes into macrophages could alter resistin levels. Therefore, it is reasonable to postulate that periodontal inflammation, possibly via cytokine secretion and macrophage maturation, might influence resistin expression (25). However, more long-term interventional studies in larger sample sizes are needed to fully uncover the cause-effect relationships between resistin levels and diabetes-related periodontitis (8).

### Visfatin

Visfatin, also known as pre-B-cell colony enhancing factor, is a 52 kDa adipokine secreted by visceral adipose tissues (28). By binding to insulin receptor at a site distinct from that of insulin, visfatin exerts insulin-like effects to reduce glucose release and stimulate glucose utilization in adipocytes and myocytes (40). Furthermore, visfatin has been reported to induce the productions of proinflammatory cytokines, like IL-6, TNF-α and IL-1β, during infection and inflammation phases (29). During periodontal inflammation, periodontopathogens trigger local expressions of IL-6 and TNF-α in periodontal tissues. These proinflammatory cytokines, in turn, trigger visfatin production in periodontal tissues. Bahammam and Attia have found significantly elevated levels of IL-6, TNF-α, and visfatin in GCF of diabetic patients afflicted with chronic periodontitis (27). Clinically, compared to periodontally healthy individuals and diabetic patients, the mean visfatin levels remained the highest in both serum and GCF of diabetic patients afflicted with chronic periodontitis. Meanwhile, visfatin concentrations in both serum and GCF were shown positively correlated with the severity of periodontal disease (30). Importantly, non-surgical periodontal treatment was reported to remarkably reduce visfatin levels in serum and GCF of diabetic patients with periodontitis (31). These clues suggested that visfatin might serve as a potential predictor and therapeutic target in the management of diabetes mellitus and periodontitis (41).

## Proinflammatory cytokines

It is well established that diabetes is a disorder of inflammation and metabolic dysregulation, associated with increased production of cytokines, including IL-6, IL-1β, and TNF-α (42, 43). It has been suggested that periodontal therapy could reduce systemic inflammation in diabetic patients by targeting intraoral bacteria and reducing periodontal inflammation (44). Poor glycemic control in type 2 diabetic patients is clinically associated with poor prognosis of periodontal tissues (45). Levels of these inflammatory cytokines are significantly higher in patients with periodontitis (46). These cytokines stimulate bone resorption by inducing osteoclast progenitor proliferation, as well as the production of chemokines, extracellular matrix metalloproteinases (MMPs), cytokines, collagenases, and prostaglandins (47). Besides, increased levels of these cytokines further alter insulin sensitivity through direct and indirect mechanisms (48), resulting in a vicious cycle between diabetes progression and periodontal damage (49). Interestingly, periodontal therapy could elicit beneficial effects on glycemic control via suppressing these cytokines, facilitating a less-pronounced inflammatory state (43, 50). Moreover, IL-6, IL-1β, and TNF-α are confirmed important modulators in bone metabolism within oral cavity. Altogether, these cytokines in serum and GCF can be potentially considered as biomarkers in the prediction, intervention and treatment of chronic periodontitis afflicted with type 2 diabetes mellitus (43, 49, 51).

Another important mediator in chronic inflammatory diseases, soluble tumor necrosis factor-like weak inducer of apoptosis (sTWEAK), is gaining increasing attentions recently (52). sTWEAK belongs to the TNF superfamily cytokines and elicits an immunoregulatory role in periodontitis and diabetes mellitus (53, 54). Serum sTWEAK level was shown to be significantly lower in patients with chronic periodontitis, and lowest in patients with concomitant chronic periodontitis and type 2 diabetes mellitus (55). However, another recent clinical study found that significantly higher levels of circulating sTWEAK were observed in severe periodontal patients when compared to those without periodontitis (56). More extensive studies are still needed to correlate the temporal change in circulating sTWEAK level with the progression of concomitant periodontitis and diabetes mellitus.

## Oxidative stress markers

Increasing oxidative stress can be considered as a critical contributing factor during the pathogenesis of both diabetes mellitus and periodontitis (57). Some previous clinical studies have reported the alterations of oxidative stress markers in different body fluids of patients with concomitant periodontitis and diabetes mellitus. For instance, the salivary levels of the free radical marker malondialdehyde were higher in patients with chronic periodontitis when compared to healthy individuals, where the levels became even higher in patients with concomitant periodontitis and diabetes mellitus (58). Superoxide dismutase (SOD) is an antioxidant enzyme which protects against deleterious effects of high oxidative stress. Another clinical study has shown that the serum levels of superoxide dismutase were the highest in patients with concomitant periodontitis and diabetes mellitus when compared to those of periodontal patients and healthy individuals (59). Catalase is one the central antioxidant enzymes to constitute the primary defense against oxidative damage. A previous clinical trial found that the serum levels of catalase in patients with concomitant periodontitis and diabetes mellitus were lower than individuals with and without periodontitis (60). Furthermore, the serum and GCF levels of 4-hydroxy-2-nonenal, a product of lipid peroxidation, were shown to be higher in patients with both diabetes mellitus and periodontitis (61). These clinical findings suggested oxidative stress as a link between the two diseases.

## MicroRNAs

MicroRNAs represent a group of small non-coding regulatory RNAs (~22 nucleotides) which post-transcriptionally lower stability and suppress gene expression (62). In mammalian cells, more than 2,500 microRNAs have been reported to regulate >60% of protein-coding genes (63). Revealed by extensive studies, microRNAs have been confirmed to regulate various physiological and pathological processes in various human diseases, including autoimmune disorders, cancers and inflammatory disorders (64). Of note, microRNAs play a regulatory role in the pathogenesis of periodontitis, where the microRNA profiles in healthy and inflamed gingival tissues significantly vary (65, 66). Meanwhile, microRNAs also play a critical role in the mediation of glucose homeostasis and progression of diabetes mellitus. Nowadays, more and more studies have recognized microRNAs as biomarkers in clinical medicine, where microRNAs can potentially be prognostic and predictive biomarkers in the treatment of chronic periodontitis and diabetes mellitus (67–69) (**Table 2**).

**Table 2:** MicroRNAs as potential biomarkers for periodontitis and diabetes mellitus.

microRNAs	Biological functions	Correlation with periodontitis	Correlation with diabetes mellitus	Ref
miR-146a	· Mediates the pathogenesis of multiple inflammation-associated disorders (e.g. periodontitis, diabetes mellitus and coronary artery disease)	· Increased in gingival tissues during periodontitis · Inversely correlated to proinflammatory cytokine levels (e.g. TNF-α and IL-6) · Non-surgical periodontal treatment reduces its GCF level	· Decreased in peripheral blood mononuclear cells and serum during type 2 diabetes mellitus · Inversely correlated to proinflammatory cytokine levels (e.g. IL-8) · Increased in GCF during diabetes-related periodontitis	(69–73)
miR-214	· Regulates cell death · Regulates glucose metabolism	· Activates necroptosis in periodontal tissues and osteoblast cells	· Inversely correlated to obesity and insulin resistance	(74–77)
miR-147	· Attenuates inflammatory response · Mediates macrophage activation	· Promotes macrophage activation in periodontal tissues	· Inversely correlated to proinflammatory cytokine expression in macrophages (e.g. TNF-α and IL-6)	(78, 79)
miR-126	· Regulates inflammatory cytokine secretion	· Suppresses inflammation in gingival fibroblasts · Positively correlated to anti-inflammatory cytokine levels (e.g. IL-10)	· Inversely correlated with diabetes mellitus	(80)

*Red color: body fluids or tissues/cells where these biomarkers could be collected for potential diagnostic purpose.

Various microRNAs have been demonstrated to be key regulators in inflammation. A substantial literature indicated that miR-146a is involved in the pathogenesis of multiple inflammatory disorders, such as chronic periodontitis, diabetes mellitus and coronary artery disease (70). miR-146a levels have been found significantly lower in peripheral blood mononuclear cells from patients with type 2 diabetes mellitus (71). Notably, serum levels of miR-146a remarkably decrease in type 2 diabetic patients, which is inversely correlated to that of the proinflammatory cytokine IL-8 (72). Moreover, the miR-146a expression also changes during chronic periodontitis. Motedayyen et al. demonstrated that higher levels of miR-146a, whereas lower expressions of inflammatory cytokines, particularly IL-6 and TNF-α, were observed in the gingival tissues of periodontitis patients (73). These findings suggested miR-146a as a negative regulator for immune response (81). Since miR-146a plays crucial roles in both diabetes mellitus and periodontitis, it is reasonable to hypothesize that miR-146a may participate in the bidirectional relationship of two diseases. A recent study has revealed that expressions of inflammatory cytokines diminished upon transfection of miR-146a into lipopolysaccharides (LPS)-stimulated adipocytes and gingival fibroblasts, when co-cultured with macrophages. Similarly, transfection of miR-146a into macrophages down-regulated TNF-α expression in the presence of inflammatory stimuli. *In vivo* findings suggested that intravenous injection of miR-146a protected C57BL/6 mice from high-fat diet-induced inflammatory insults in adipose and gingival tissues. These findings implied a protective role of miR-146a against inflammation-related obesity and periodontal disease (82). Clinically, miR-146a level was reported to be significantly higher in GCF of type 2 diabetic patients afflicted with periodontitis, but decreased upon non-surgical periodontal treatment (69). These preclinical and clinical studies hinted that miR-146a might be a potential biomarker and therapeutic target for the treatment of periodontitis and diabetes mellitus.

Other microRNAs, including miR-214, miR-147 and miR-126, have been reported to play pivotal roles in both diabetes and periodontitis. Necroptosis, a newly discovered mode of programmed cell death, is a highly proinflammatory event involved in the pathogenesis of periodontitis and diabetes (83, 84). Previous studies have shown that miR-214 is responsible for the regulation of cell death and glucose metabolism (74–76). Ou et al. has found that miR-214 regulates necroptosis through targeting activating transcription factor 4 (ATF4) in periodontal tissues and osteoblast cells under co-stimulation by high glucose and LPS (77). In rats, experimental periodontitis was shown to promote systemic insulin resistance by inducing macrophage activation and hence inflammation in adipose tissue (85). Notably, miR-147 was reported to act as a negative regulator in attenuating inflammatory response in murine macrophages (78). However, another study showed that miR-147 seems to promote M1 polarization, the classical activation of macrophages, in periodontal tissues of obese rats (79). Further verification and mechanistic study are required to uncover the conflicting role of miR-147 in periodontitis and diabetes mellitus, especially in human subjects. Another microRNA, miR-126, has been shown to play a protective role against high glucose-induced inflammation in human gingival fibroblasts. Mechanistically, miR-126 promotes the secretion of the anti-inflammatory cytokine IL-10 via targeting tumor necrosis factor (TNF) receptor associated factor 6 (TRAF6) (80). Taken together, the emerging roles of microRNAs, including miR-146a, miR-214, miR-147 and miR-126, may provide new insights into the diagnostic and therapeutic strategies on the treatment of concomitant periodontitis and type 2 diabetes mellitus.

## Glycoproteins

A chitin- and heparin-binding glycoprotein, YKL-40, is secreted by activated neutrophils and macrophages during acute or chronic inflammatory diseases (86). Among patients with chronic periodontitis, GCF levels of YKL-40 in patients with type 2 diabetes mellitus were often higher (87–89). Another acidic glycoprotein, Chromogranin A (CgA), is identified in the extracellular vesicles secreted by neurons and endocrine cells. The concentration of CgA is known to be increased in response to psychological stress, the risk factor for periodontal disease (90, 91). Zhang et al. indicated that CgA values in saliva samples of chronic periodontitis patients with or without type 2 diabetes mellitus were significantly higher than those of control groups. These findings suggested that salivary CgA could be a potential biomarker for periodontitis and diabetes mellitus (92).

## Oral microbes

Our oral cavity is inhabited by diverse microbes including bacteria, fungi and protozoa, where over 700 bacterial species have been identified (93). Disruption of balance between commensal and harmful microbes in the oral cavity is associated with the pathogenesis of certain diseases, including periodontitis (94), diabetes mellitus (95), and cancers (96). In addition to the endogenous biomarkers mentioned, such as adipokines and microRNAs, the microbes in the oral cavity can also be potential biomarkers of multiple diseases. Notably, alteration in the abundance of certain bacterial species in the oral cavity may be indicative to the severity of periodontitis and diabetes mellitus (**Table 3**).

**Table 3:** Oral bacteria as potential biomarkers for periodontitis and diabetes mellitus.

Oral bacteria	Effects on host	Correlation with periodontitis	Correlation with diabetes mellitus	Ref
*P. gingivalis*	· Forms subgingival biofilm (increased DPPIV activity) · Induces the production of proinflammatory cytokines (IL-1β and IL-6) · Enhances osteoblast activation and hence bone resorption	·Salivary concentrations positively correlated with periodontitis	· Inhibits host glucose homeostasis (glycogen synthesis and insulin resistance) · Periodontal treatment reduces *P. gingivalis* level and improves glycemic profiles	(97–103)
*F. Nucleatum*	· Acts as intermediate colonizer bridging the attachment of commensal and pathogenic bacteria · Facilitates a reducing microenvironment for colonization of anaerobes · Promotes recruitment of macrophages and osteoclasts to gingival tissues	· Positively correlated with periodontitis	· Increased subgingival levels in patients with uncontrolled type 2 diabetes mellitus · Increased abundance in patients with both periodontitis and diabetes mellitus	(104–108)
*T. forsythia*	· Triggers destruction of connective tissue · Promotes resorption of alveolar bone · Facilitates bacterial adhesion · Promotes the production of inflammatory cytokines (e.g. IL-6 and IL-10)	· Positively correlated with periodontitis	· Increased subgingival levels in diabetic patients · Positively correlated to resistin levels · Positively correlated to serum SAA levels	(109–114)
*P. nigrescens*	· Shifts from commensalism to virulence · Upregulates expression of MMPs · Induces the production of proinflammatory cytokines	· Positively correlated with periodontitis · Periodontal treatment reduces *P. nigrescens* level, and IL-6 level in serum and GCF	· Increased abundance in diabetic patients	(115–120)

*Red color: body fluids or tissues/cells where these biomarkers could be collected for potential diagnostic purpose.

### Porphyromonas gingivalis

*Porphyromonas gingivalis (P. gingivalis)* is a Gram-negative anaerobe in the oral cavity, and is considered as the major pathogenic bacterium for periodontitis (121). The capacity of *P. gingivalis* to form subgingival biofilm, associated with increased DPPIV activity, greatly contribute to the pathogenesis of periodontitis (98). Additionally, *P. gingivalis* promotes pathogenesis of aggressive periodontitis by inducing the production of proinflammatory cytokines, such as IL-1β and IL-6, from peripheral T helper cells (99). Meanwhile, *P. gingivalis* can enhance osteoclast activation and Th1-Th17-response, which further aggravate bone resorption during the pathogenesis of destructive periodontitis (103). Clinically, the salivary concentrations of *P. gingivalis*, IL-1β and matrix metalloproteinase (MMP)-8 are associated with the severity of periodontitis (102). On the other hand, *P. gingivalis* overgrowth in the oral cavity can affect host glucose homeostasis. Once oral *P. gingivalis* is translocated to the liver, it can inhibit glycogen synthesis via the Akt/GSK-3β signaling, resulting in a higher glucose level (100). Oral colonization with periodontal pathogens, particularly *P. gingivalis*, impaired insulin resistance in high fat diet-fed mice (97). In a previous clinical study, periodontal treatment improved glycemic profiles and reduced detection rate of subgingival *P. gingivalis* in type 2 diabetic patients (101). These preclinical and clinical findings suggested *P. gingivalis* as a potential microbial biomarker for periodontitis and diabetes mellitus.

### Fusobacterium nucleatum

Another periodontal pathogen, *Fusobacterium nucleatum (F. Nucleatum)* is also correlated to the occurrence of diabetes mellitus. *F. Nucleatum* is a Gram-negative anaerobe, predominantly found in biofilms of dental plaques (122). *F. Nucleatum* acts as an intermediate colonizer bridging the attachment of commensal and pathogenic bacteria on tooth and epithelial surfaces. Moreover, *F. Nucleatum* contributes to the reducing microenvironment that facilitates the colonization of oxygen-intolerant microbes (106). Coherently, *F. Nucleatum* significantly enhanced the invasion of human gingival epithelial cells by *P. gingivalis* (108). Oral infection with *F. Nucleatum* promotes recruitment of macrophages and osteoclasts towards gingival tissues, driving inflammation and bone resorption (105). Clinically, among patients suffering from chronic periodontitis, higher subgingival levels of *F. Nucleatum* were observed in those with uncontrolled type 2 diabetes mellitus (104). Meanwhile, higher *F. Nucleatum* levels were associated with poorer glycemic control in patients with both chronic periodontitis and type 2 diabetes mellitus (107). However, the detailed mechanism on how *F. Nucleatum* is related to the progression of diabetes mellitus remains elusive.

### Tannerella forsythia

*Tannerella forsythia (T. forsythia)*, another Gram-negative anaerobe, is also considered as a major contributor to the development of periodontitis. *T. forsythia* triggers destruction of connective tissue and resorption of alveolar bone during periodontitis progression (110). Higher subgingival *T. forsythia* levels were observed in obese individuals than those with normal BMIs (111), implying a higher risk of periodontitis in obese individuals. The leucine-rich-repeat protein (BspA) expressed by *T. forsythia* has been shown to play a contributory role in bacterial adhesion and inflammation in dental tissues. *T. forsythia* can stimulate the secretion of proinflammatory cytokines and chemokines from monocytes and osteoblasts respectively, driving inflammation and bone resorption (113). Additionally, *T. forsythia* enhances the expression of inflammatory cytokines (e.g. IL-6 and IL-10) in macrophages and dendritic cells, in a toll-like receptor 2 (TLR2)-dependent manner (112). In type 2 diabetic patients, the abundance of *T. forsythia* in subgingival plaque was found higher than that of non-diabetic individuals (114). Interestingly, higher levels of periodontal pathogens, including *P. gingivalis* and *T. forsythia*, were observed along with higher resistin levels in saliva of obese type 2 diabetic patients (109). In mice, oral infection with *T. forsythia* remarkably increased the serum levels of serum amyloid A (SAA), the subclinical inflammatory biomarker in multiple diseases like diabetes mellitus, atherosclerosis and rheumatic diseases (110).

### Prevotella nigrescens

*Prevotella nigrescens* (*P. nigrescens*) is another Gram-negative and non-spore forming anaerobe commonly found in the dental plaques of periodontitis patients (118). During the progression of periodontitis, *P. nigrescens* shifts from commensalism to virulence via the upregulation of MMPs (120). High levels of MMPs (e.g. MMP-8 and MMP-9) often reflect periodontal inflammation (115). *P. nigrescens* can induce IL-1β production in dendritic cells through the activation of TLR2 and nucleotide-binding oligomerization domain like receptor pyrin domain containing 3 (NLRP3) inflammasome (117). In type 2 diabetic patients, higher abundance of *P. nigrescens* was also noted in periodontitis sites when compared with those of non-diabetic individuals (116). In non-diabetic pregnant women, periodontal therapy remarkably reduced *P. nigrescens* abundance in dental plaque, and IL-6 levels in serum and GCF (119). Therefore, it is reasonable to postulate that periodontal treatment may elicit similar anti-inflammatory effects in diabetic patients by decreasing *P. nigrescens* abundance.

Due to the ease of obtaining samples from saliva and dental plaques, microbes in the oral cavity might be efficient and potential biomarkers for disease diagnosis and evaluation of therapeutic outcomes in periodontitis and diabetes mellitus. However, selection of a single bacterial strain may not most accurately evaluate the severity of diseases. In contrast, selection of multiple microbial biomarkers, or even in combination with other endogenous biomarkers, may further improve the accuracy and consistency of disease prediction and evaluation.

## Therapeutic insights and future perspectives

Notably, some of the mentioned biomarkers might also serve as therapeutic targets for the treatment of diabetes mellitus and periodontitis. In other words, therapeutic strategies that could suppress the levels of certain biomarkers might alleviate the progression of the two diseases, particularly when certain biomarkers are involved in the pathogenic mechanisms of both diseases. For instance, non-surgical periodontal treatment could reduce serum and GCF levels of visfatin and improve glucose homeostasis in patients with concomitant periodontitis and diabetes mellitus (22). Of note, visfatin can alter glucose metabolism and promote inflammation in gingival tissues (123). Furthermore, periodontal treatment was shown to improve glycemic profiles along with reduced persistence of *P. gingivalis* in type 2 diabetic patients (101), where *P. gingivalis* is the major pathogenic bacterium for periodontitis and can alter glucose level once translocated to liver (100). Future studies shall investigate whether other biomarkers participate in the pathogenic processes of both diseases for more therapeutic insights.

The above studies also indicate that periodontal treatment is possible to alter glycemic control in patients. On the other hand, treatment that improves glycemic control might promote periodontal health. A previous clinical study showed that effective glycemic control without periodontal treatment could also improve bleeding on probing in patients (124). It is therefore interesting to investigate whether glycemic control alone could alter biomarker levels in oral cavity (e.g. GCF) of patients in future study. Besides, further efforts are needed to clarify whether a certain biomarker is a cause or consequence of diabetes mellitus and periodontitis. Therapeutic strategies that alter the consequent biomarker might not necessarily alleviate lesions and disease progressions of both diseases.

## Conclusions

Epidemiologically, periodontitis is now considered as a risk factor for diabetes mellitus, and has been designated as the sixth complication of diabetes mellitus (125, 126). It is also reasonable to consider periodontitis as a co-morbidity of diabetes mellitus (127). Increased periodontal breakdown in patients with diabetes can attribute to the activation of immune and inflammatory responses, and increased susceptibility to infection. Certain endogenous biomarkers, including adipokines, microRNAs, inflammatory mediators, oxidative stress markers, and glycoproteins have been reported to play important roles in initiating and regulating different effector stages of immune and inflammatory responses (69, 128). Potentially, these low-molecular-weight proteins or non-coding RNAs are not only therapeutic targets, but also clinical predictors for earlier diagnosis and intervention for periodontitis and diabetes mellitus (20, 129). Importantly, overgrowth of certain bacterial strains in the oral cavity might be indicative to both periodontitis and diabetes mellitus. Further biomarker research would be a worthwhile endeavor to deepen our understanding towards the bidirectional relationship between type 2 diabetes mellitus and periodontitis.

## Author contributions

SL: Writing – original draft. HL: Investigation, Validation, Writing – review & editing. HK: Writing – original draft. SYW: Investigation, Validation, Writing – review & editing. CKC: Conceptualization, Writing – review & editing. JX: Funding acquisition, Supervision, Writing – review & editing.
