# Supplementary figures and images for: Endogenous and microbial biomarkers for periodontitis and type 2 diabetes mellitus
Source: Front Endocrinol (Lausanne). 2023 Dec 5;14:1292596. doi: 10.3389/fendo.2023.1292596 (PMC10750125; doi:10.3389/fendo.2023.1292596)

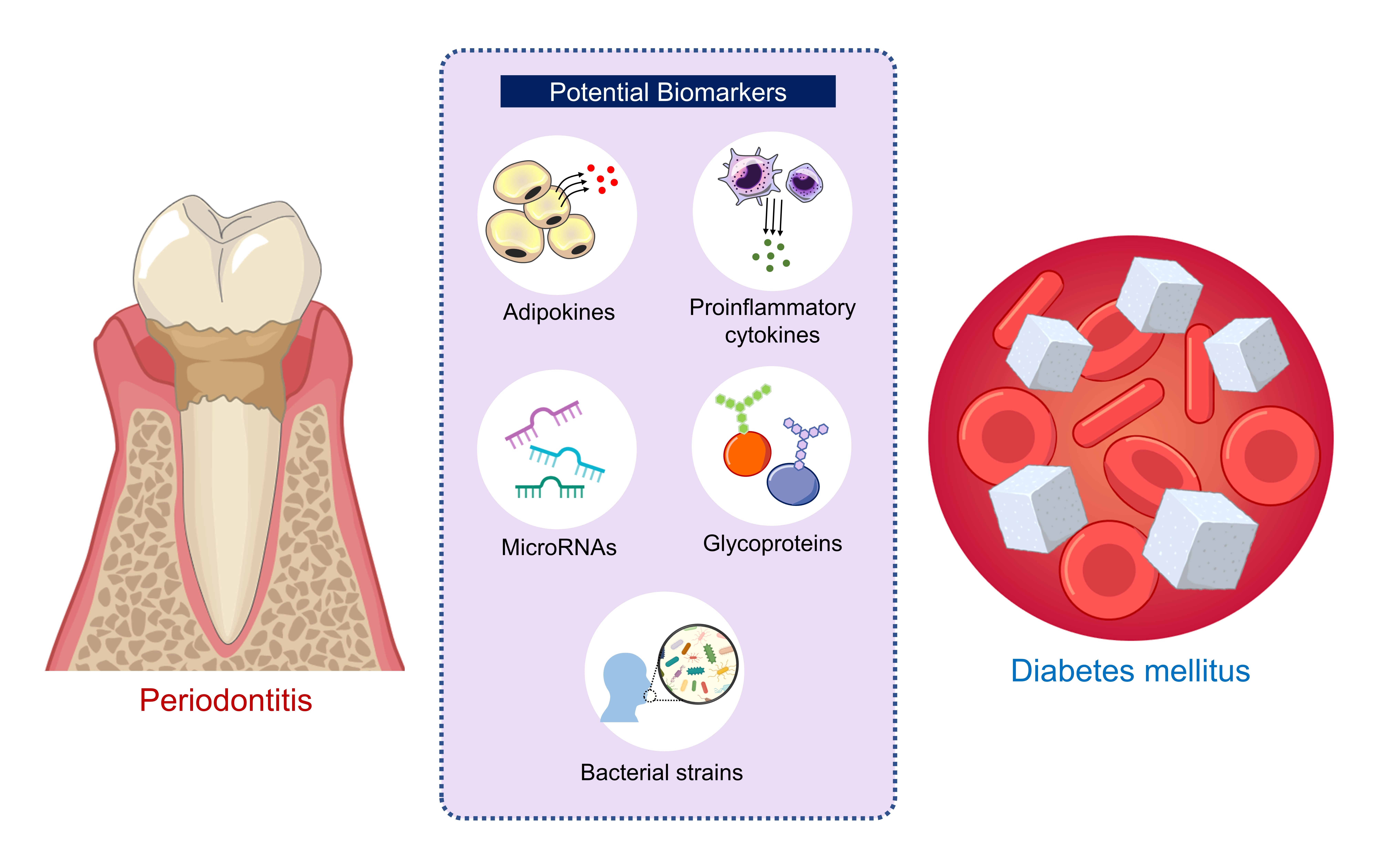

Supplement: Supplementary file 1 [file Image_1.jpeg]
